# Improved reannotations of *Aegilops umbellulata* (PI 554389) genome and transcriptomics data provide candidates for leaf rust resistance for wheat improvement

**DOI:** 10.1038/s41598-025-29241-6

**Published:** 2025-12-29

**Authors:** Jatinder Singh, Santosh Gudi, Peter J. Maughan, Rajeev Gupta, Upinder Gill

**Affiliations:** 1 North Dakota State University, Fargo, ND USA; 2 Brigham Young University, Provo, UT USA; 3 United States Department of Agriculture (USDA) Agricultural Research Service (ARS), Cereal Crops Improvement Research Unit, Edward T. Schafer Agriculture Research Center, Fargo, ND USA

**Keywords:** Aegilops umbellulata, Leaf rust, Puccinia triticina, Reannotations, Resistance gene analogs, Wheat wild relative, Computational biology and bioinformatics, Genetics, Molecular biology, Plant sciences

## Abstract

Leaf rust caused by *Puccinia triticina* is a yield limiting disease of cultivated wheat throughout the world. *Ae. umbellulata* is a diploid species of wheat wild relatives containing high genetic diversity for resistance to multiple races of leaf rust. Here, we used short-read data along with long-read transcript data to reannotate (version 2) the *Ae. umbellulata* acc. PI 554389 reference genome. In this annotation, we reported 62,434 gene models consisting of 41,906 high confidence (HC) gene models and 20,992 low confidence (LC) gene models. Comparative transcriptome profiling of resistant (PI 554389) and susceptible (PI 554417) accessions challenged with TNBJS revealed distinct temporal patterns of defense activation. The resistant accession exhibited an early and stronger transcriptional response at 6 h after inoculation, whereas the susceptible accession showed delayed expression changes. In comparisons between treated and mock samples of the resistant accession, we identified two candidate resistance genes—one encoding a leucine-rich repeat and another with a kinase domain—that were differentially expressed at early infection stages, suggesting potential roles in pathogen recognition or downstream signaling. This integrative genomic and transcriptomic framework enhances the functional characterization of *Ae. umbellulata* and identifies promising candidate genes for functional validation and deployment in wheat improvement programs aimed at achieving durable leaf rust resistance.

**Supplementary Information:**

The online version contains supplementary material available at 10.1038/s41598-025-29241-6.

## Introduction

Wheat provides 20% of all calories consumed by humans worldwide. Global wheat demand is projected to increase due to increasing population^1^. Plant breeding efforts in the past few decades have been focused on increasing wheat yield, but fungal pathogens continue to be a major impediment of wheat production. Global yield losses due to biotic factors are estimated to range from 20 to 40% annually, depending on the region and year^2^. Three wheat rusts – stem rust, stripe rust, and leaf rust (Lr) are among the major biotic factors limiting yield. Among three rusts, Lr is widely distributed and found commonly in almost all wheat growing areas. Under conducive weather conditions, the yield losses due to Lr can be upto 50%, causing severe epidemics^3^. Indeed, Lr is reported to cause the largest crop losses in wheat globally among all the wheat pathogens^2^. It is caused by a dikaryotic, macrocyclic, obligate biotrophic fungus, *Puccinia triticina* Eriks. (*Pt*). Owing to its heteroecious nature, *Pt* requires an alternate host (*Thalictrum* spp.) to complete its sexual life cycle, and infects wheat only at its uredial stage. Sexual recombination and natural mutations lead to increased genetic diversity in *Pt* populations, which results in the appearance of new *Pt* races^4^. In the absence of sexual reproduction, nuclear exchange between the leaf rust races also leads to emergence of new and diverse leaf rust races^5^. This is especially true in case of emergence of novel races in the United States due to the lack of an alternate host for sexual reproduction^6^. It is predicted that changing weather patterns will worsen the pathogen severity by decreasing the latency period, which in turn will increase the number of infection cycles and sporulation efficiency^7^. On average, 30–60 different *Pt* races are reported every year in the United States - significantly higher compared to other wheat rust pathogens^8^. Leaf rust is widely adapted, occuring in almost all continents where wheat is cultivated^3^. Owing to its rapid adaptation, virulent *Pt* races quickly render leaf rust resistance genes ineffective leaving cultivated wheat susceptible to significant yield losses.

The recent polyploidization and extensive domestication of bread wheat have resulted in reduced genetic diversity, particularly in loci associated with disease resistance^9^. Enhancing genetic diversity in wheat is essential to counter the ongoing threats of pathogen evolution and changing weather patterns. There are more than 80 *Lr* resistance genes that have been characterized and nearly half of them have been identified from secondary and tertiary gene pool of wheat^10,11^. Wheat wild relatives (WWRs) are a rich reservoir of resistance genes and many species have been extensively mined for Lr resistance genes^9,12^. The first leaf rust resistance gene ever introgressed from WWRs to the cultivated wheat was *Lr9* which was transferred from *Aegilops umbellulata* (Sears 1965). *Lr9* has been recently cloned and it encodes a tandem kinase protein, which provides race specific resistance to leaf rust^13^. In addition to *Lr9*, another leaf rust resistance gene (*Lr76*; located on chromosome 5DS) has also been transferred from *Ae. umbellulata* to hexaploid wheat^14,15^. *Ae. umbellulata* harbors high genetic diversity^16^ and has been reported to harbor leaf rust resistance genes for multiple Lr races^17^, nonetheless it has been under-utilized for trait transfer to wheat. Out of 11 cloned *Lr* genes, seven (*Lr1*,* Lr10*,* Lr13*,* Lr21*,* Lr22a*,* Lr42*, and *Lr47*) are classified as nucleotide binding-leucine-rich repeat receptors (NLRs) proteins. A recently study suggests that the functional NLRs express throughout the plant tissues regardless of pathogen infection^18^.

With the advancements in sequencing technologies, RNAseq studies become affordable and are being used to elucidate the molecular mechanisms of gene functions. Transcriptomics analysis provides an efficient approach to discover genes associated with plant defense^18^. While genome assemblies provide the structural framework, the annotations reveal the functional landscape of the genome. Unlike purely genomic comparisons, transcriptomic profiling captures the dynamic regulation of defense responses following pathogen attack, enabling rapid identification of genes that are activated or suppressed during infection^19^. Differentially expressed genes (DEGs) often include receptors, kinases, and signaling components involved in effector recognition and defense activation^20^. In the current study, we presented an improved annotations of the recently published reference genome of *Ae. umbellulata*^17^ to provide more accurate gene models and resistance gene analog prediction. Further, we used the transcriptomics data from two contrasting *Ae. umbellulata* accessions to determine the underlying mechanism of leaf rust resistance and to identify candidate R genes for further characterization.

## Results

### Updated gene annotations of *Ae. umbellulata* reference genome of PI 554389

**Fig. 1:**
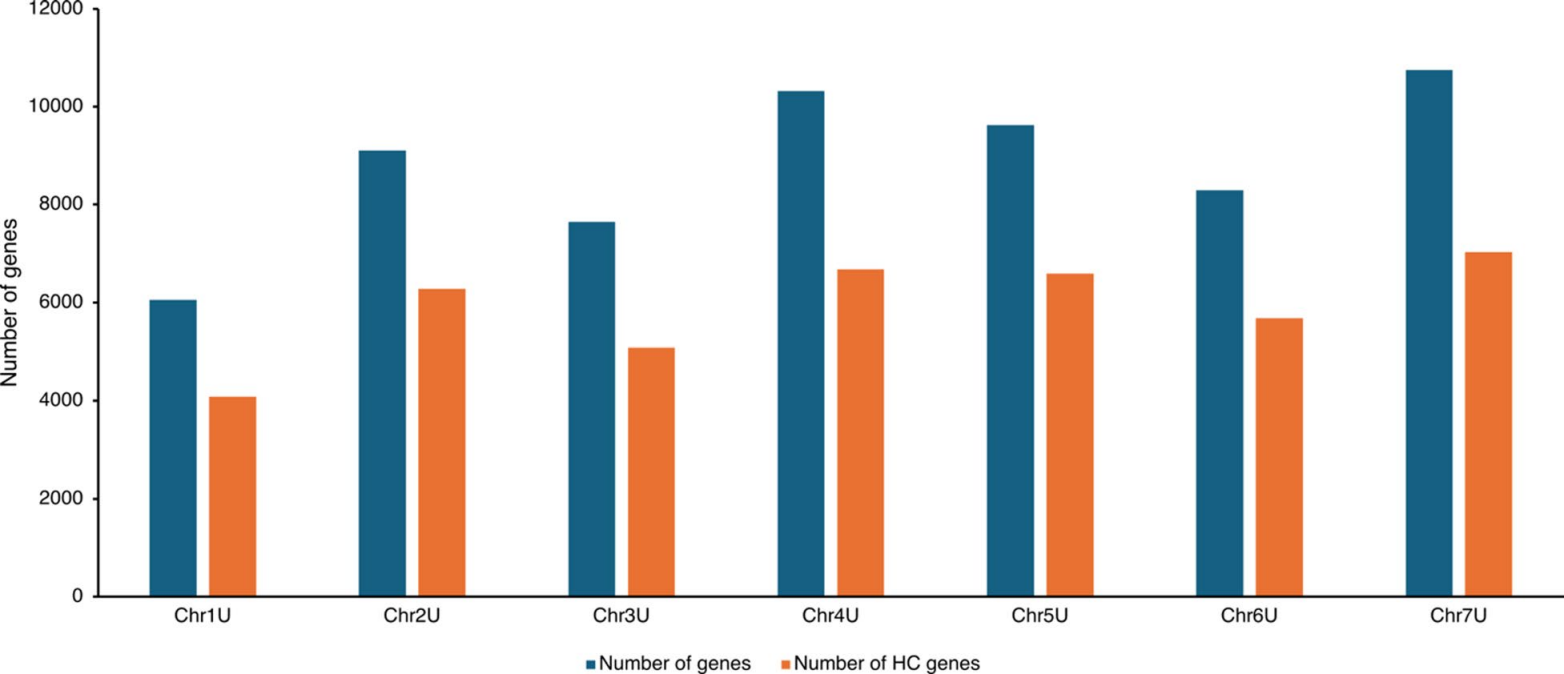
Distribution of the total number of genes and high confidence (HC) genes on seven *Ae. umbellulata* chromosomes.

To refine the gene models of the reference genome of *Ae. umbellulata* acc. PI 554389^17^, we used short read RNAseq and long read PacBio IsoSeq data to generate a more accurate and comprehensive set of gene models using the BRAKER3 annotation pipeline^21^. In the revised annotations (v2), we annotated 62,434 gene models with 70,433 mRNA transcripts. The completeness of the annotated gene model was measured using BUSCO score, which suggests highly complete gene models with 98% completeness for poales (poales_odb10). Further classification identified 41,906 high confidence (HC) gene models (47932 transcripts) and 20,992 low confidence (LC) gene models (22501 transcripts) (Fig. 1, Table S1).

The revised annotations (v2) reported lower number of gene models compared to the first version (v1) of annotation of *Ae. umbellulata* acc. PI 554389 reference genome^17^. In v1, there were 78,076 gene models with 48,366 and 29,710 classified as HC and LC gene models, respectively. In v2, the single exon genes were reported to be 13,277 in HC gene models and 12,230 in LC gene models (Table S2). Whereas in v1, there were 14,179 and 15,684 single exon genes in HC and LC gene models, respectively. Further, the multi exon genes were reported to be significantly lower in v2 than v1 (Table 1). We annotated 28,629 (HC) and 8762 (LC) multi exon genes in v2 in comparison to 34,187 (HC) and 14,026 (LC) multi exon genes in v1. We also noticed a difference in the number of exons per gene and the average exon length in the improved version (v2) as compared to previous version (v1). For instance, there are four exons per gene in v2 with an average 289 bp per exon, whereas in v1, there were three exons per gene with an average 344 bp per exon. The average number of genes per megabase (Mb) was reported to be highest on Chr4U (19), followed by Chr7U (16) and lowest were on Chr1U (12) (Table S3).

**Table 1:** Comparison of *Aegilops umbellulata* acc. PI 554389 annotations between version 1 (v1) and version 2 (v2).

	*Ae. umbellulata* acc. PI 554389 v1			*Ae. umbellulata* acc. PI 554389 v2		
	Total genes	HC genes	LC genes	Total genes	HC genes	LC genes
Genes	78,076	48,366	29,710	62,434	41,906	20,992
Transcripts	78,076	48,366	29,710	70,433	47,932	22,501
Exons	265,177	195,732	69,445	250,154	210,232	39,922
Average gene length	2316	2752	1606	2094	2681	931
Average CDS length	1021	1167	784	1026	1220	612
Average exon length	344	338	363	289	278	345
Average Intron length	478	477	566	522	534	417
Exons per gene	3	4	2	4	5	2
Exons per transcript	3	4	2	4	4	2
Monoexonic genes	29,863	14,179	15,684	25,082	13,277	12,230
Monoexonic transcripts	29,863	14,179	15,684	28,936	15,214	13,722
Multiexonic genes	48,213	34,187	14,026	37,352	28,629	8762
Multiexonic transcripts	48,213	34,187	14,026	41,497	32,718	8779

### Resistance gene analogs (RGAs) in gene annotations v2

The RGAs were predicted in v2 using RGAugury that resulted in the identification of 2310 RGAs in the reannotated gene models (Table S4). About 51% of the RGA signals correspond to RLK (receptor like kinase), 14% to CNL (CC-NBS-LRR), 12% to NL (NBS-LRR), and remaining were NBS (5%), CN (5%), and RLP (6%) and TM-CC (7%). Total genes encoding for these RGAs were reported to be 1966, with Chr2U (320), Chr4U (344) and Chr7U (410) have 55% of the total RGAs. The remaining four chromosomes have RGA numbers ranging from 210 to 228 (Table S1). Despite its smaller size, Chr4U had the second-highest average number of RGAs per Mb (0.62) among all chromosomes, following Chr7U, which is approximately 100 Mb larger (Table S3).The RGAs on Chr4U were observed to be accumulated on one side of the chromosome arm.

RGAs identified in *Ae. umbellulata* exceeded those reported in *Ae. tauschii* and cultivated wheat (*T. aestivum)* (Table S5). The RGAs such as, NBS, CNL, TNL, CN, TN, NL, RLP, RLK, and TM-CC, predicted in *Ae. umbellulata* were 2304, compared to 1921 in *Ae. tauschii* and 2243 in *T. aestivum*^22^. Notably, *Ae. umbellulata* exhibited higher counts across several RGA classes, including CNL, NL, RLP, and RLK, suggesting a potentially rich repertoire of innate immune receptors in *Ae. umbellulata*.

### Transcriptomics analysis of two contrasting *Ae. umbellulata* accession for *Pt*

**Fig. 2:**
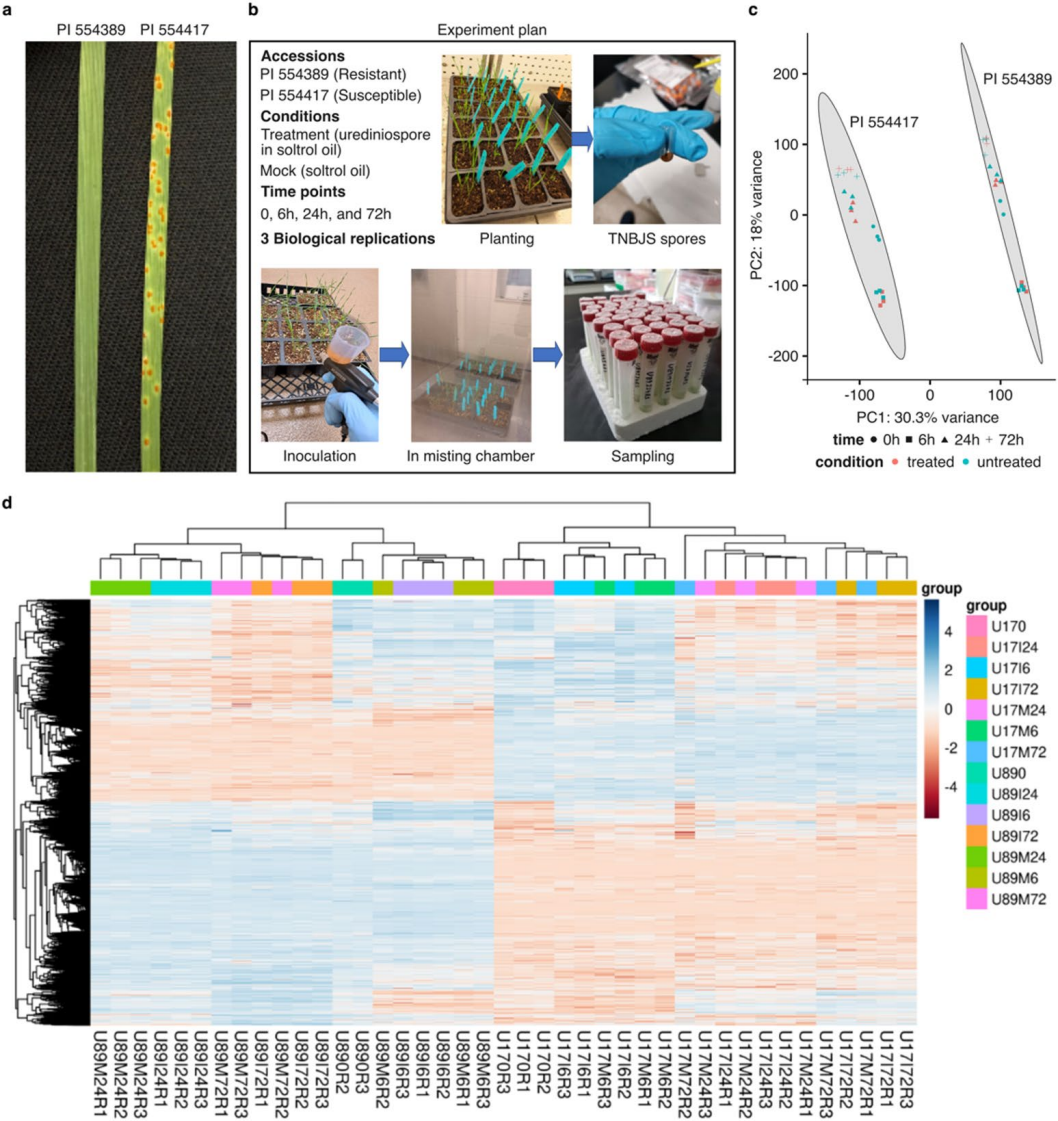
(**a**) Reaction of two contrasting *Aegilops umbellulata* accessions to leaf rust disease caused by *Puccinia triticina* race TNBJS, where PI 554389 exhibited a resistance (;1-) response and PI 554417 exhibited a susceptible (3+) response with medium to large uredinia. (**b**) The experimental design used for the transcriptomics study of leaf rust. The two contrasting accessions, PI 554389 and PI 554417, were inoculated separately with a rust urediniospore suspension in Soltrol-170 (treatment) and Soltrol-170 alone (mock), using a paintbrush sprayer. Leaf samples for RNAseq were collected at 0, 6, 24, and 72 h after inoculation (HAI). (**c**) Principal component analysis (PCA) indicates that the PI 554389 (U89) and PI 554417 (U17) samples cluster into distinct groups according to genotype, treatment (mock and treated), and time-point. (**d**) Heatmap showing hierarchical clustering based on expression profile of DEGs of all samples.

The two *Ae. umbellulata* accession, i.e., PI 554389 and PI 554417 were tested for their disease response against several *Pt* races in our previous study^17^. These accessions were phenotyped with *Lr9* virulent races (i.e., MNPSD and TNBJS) to find novel resistance genes. For the transcriptomics study, we choose the TNBJS race, which is virulent against the *Lr9* gene and showed contrasting virulence on two accessions (Fig. 2a). On the resistant accession (PI 554389), we observe flecks (;) due to a hypersensitive response as the primary infection type (IT) and few very minute pustules (1-), which is recorded as a secondary IT. On the other hand, the susceptible accession (PI 554417) showed medium to large uredinia, which we scored it 3 + IT (Fig. 2a).

### Mapping statistics of transcriptomics data

We generated 1.12 billion paired-end (PE) short reads, totaling 168 Gb data, for 14 samples (Fig. 2b, Figure S1, Table S6) used in our transcriptomics study. The samples were taken from two contrasting accessions, PI 554389 (U89) and PI 554417 (U17), under treated (I) and mock (M) conditions at four time points, specifically 0, 6, 24, and 72 h after inoculation (HAI) in three replications (R1, R2, and R3). After a quality check and adapter trimming, about 1.09 billion clean PE reads were retained for downstream analysis (Table S7). The cleaned PE reads were mapped to the *Ae. umbellulata* accession PI 554389 reference genome (Singh et al. 2024). On average across all replications, 21–28 million reads in PI 554389 and 18–26 million reads in PI 554417 were uniquely mapped on the reference genome. About 3–4% of the reads in PI 554389 and about 7–8% of the reads in PI 554417 mapped to multiple genomic locations (Table S7). The clear separation among sample groups and time points, as shown by principal component analysis (PCA) and hierarchical clustering heatmap, indicate the high quality and consistency of RNAseq data (Fig. 2c, d).

### Differential gene expression analysis of mock and *Pt*-treated contrasting *Ae. umbellulata* accessions

**Fig. 3:**
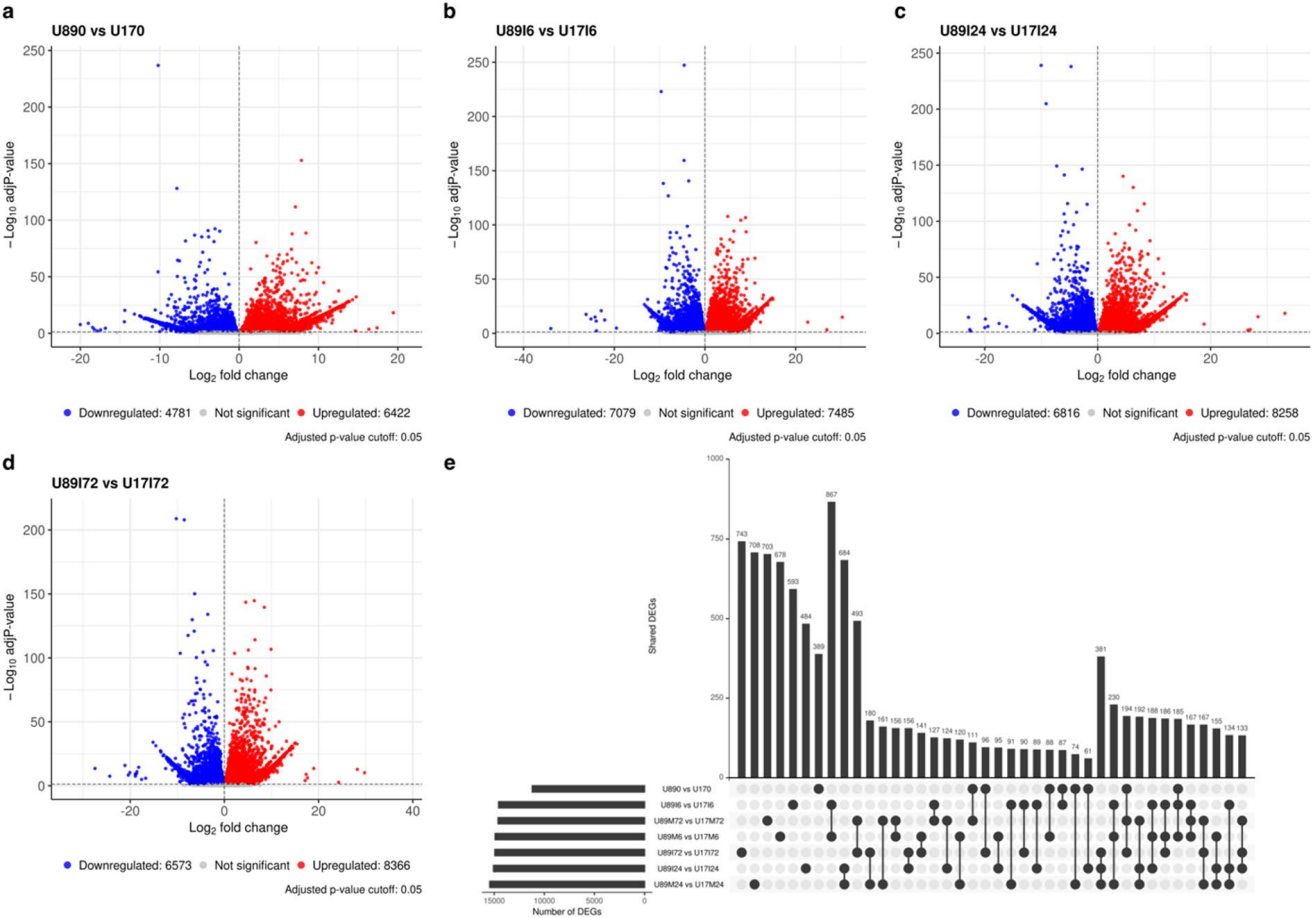
Volcano plots showing the differentially expressed genes (DEGs) classified into upregulated (red) and downregulated (blue) DEGs in Resistant (U89) vs. Susceptible (U17) comparisons at different time-points. (**a**) DEGs at 0 HAI, (**b**) DEGs at 6 HAI, (**c**) DEGs at 24 HAI, (**d**) DEGs at 72 HAI under treatment condition (rust inoculation). (**e**) Upset plot showing the shared DEGs in seven comparisons.

**Fig. 4:**
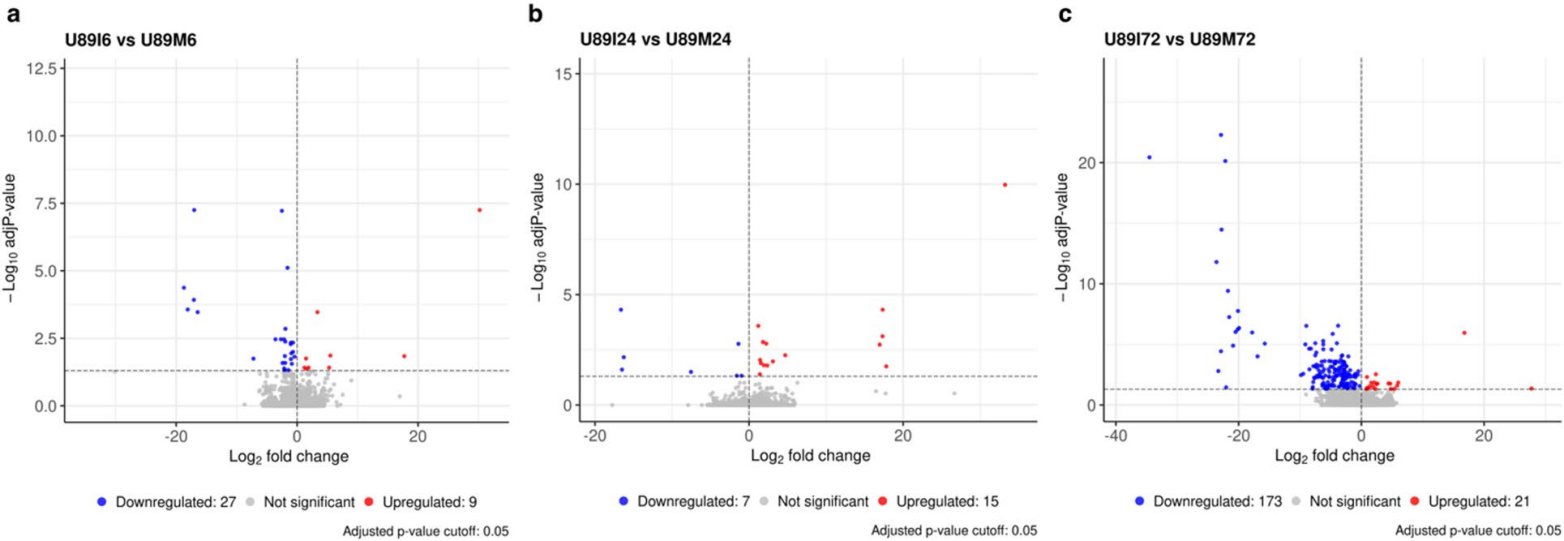
Volcano plots showing the upregulated (red) and downregulated (blue) DEGs in treatment vs. mock comparisons in resistant accession (U89) at (**a**) 6 HAI, (**b**) 24 HAI, and (**c**) 72 HAI.

**Fig. 5:**
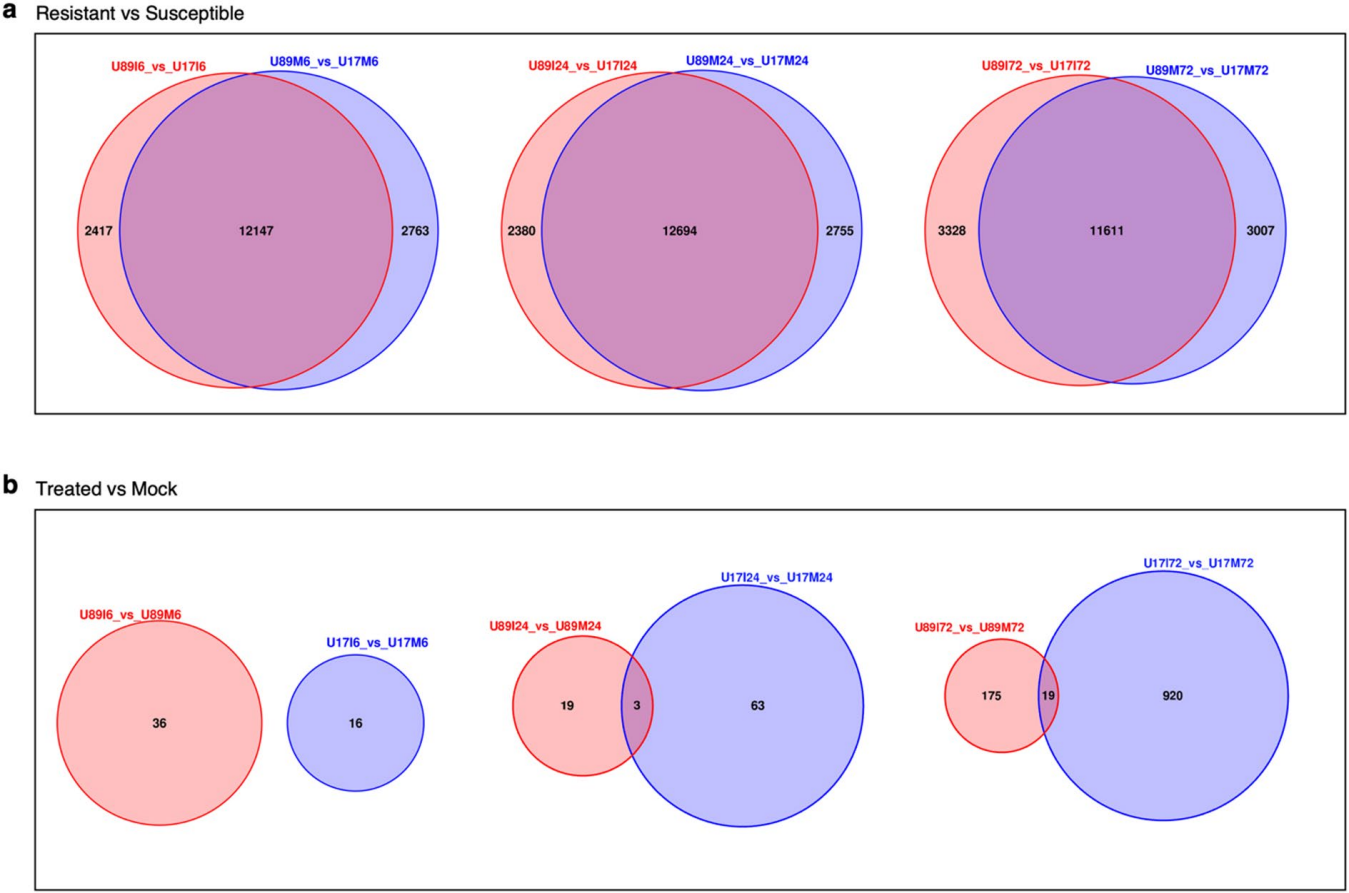
Venn diagrams showing common and unique genes in Resistant vs. Susceptible comparisons and Treated vs. Mock comparisons.

We used the DESeq2 pipeline to perform the differential gene expression analysis on the comparisons groups to study genotype (accession) effect, and treatment effect, with adjusted p-value *≤* 0.05 (Table S8). A comparison of the resistant accession (PI 554389) with the susceptible accession (PI 554417) was made to study the differentially expressed genes (DEGs) between two accessions at 0, 6, 24, and 72 HAI. The lowest number of DEGs were found at 0 h, i.e., 11,203 (6422 up-regulated and 4781 down-regulated) (Fig. 3a). These DEGs likely represent inherent genetic and transcriptional differences between PI 554389 (resistant) and PI 554417 (susceptible), reflecting their underlying genetic variation. Nearly 15,000 DEGs were reported at each of the other three time points, 6, 24, and 72 HAI, (Fig. 3b-d, Table S9). About 12,000 DEGs were common between the *Pt-*treated and mock-treated samples of the resistant and susceptible accessions comparisons (Fig. 3e). Additionally, we also compared the treatment vs. mock samples to study the *Pt* treatment effect which resulted in the identification of more DEGs in the resistant accession than in the susceptible accession at 6 HAI (Fig. 4a). But at 24 and 72 HAI, the susceptible accession showed more DEGs than the resistant accession (Figs. 4b, c, Table S8). In resistant vs. susceptible comparisons, we reported 2417, 2380, and 3328 unique DEGs in *Pt* treated samples at 6, 24, and 72 HAI (Fig. 5a). Similarly, in mock samples of resistant vs. susceptible comparisons, we found 2763, 2755, and 3007 unique DEGs at 6, 24, and 72 HAI, respectively (Fig. 5a). However, in treated versus mock comparisons, we found 0, 3, and 19 DEGs common to both accessions at 6, 24, and 72 HAI, respectively (Fig. 5b), with most DEGs being unique to each accession.

### Validation of RNAseq data by qRT-PCR

To confirm the reliability of the RNAseq based differential expression analysis, we performed quantitative real-time PCR (qRT-PCR) validation on eight randomly chosen genes by designing gene specific primers (Table S10). Total RNA from the same samples used for RNAseq was used for cDNA synthesis and gene specific primers were designed for each target gene. The qRT-PCR results showed expression patterns that were highly consistent with the RNAseq data (Figure S2), supporting the accuracy of the transcriptome profiling. In almost all cases, the direction and magnitude of gene expression changes between different comparison groups were comparable across platforms (RNAseq and qRT-PCR). This concordance reinforces the robustness of our RNAseq dataset.

### Functional analysis of DEGs in resistant vs. susceptible comparisons

**Fig. 6:**
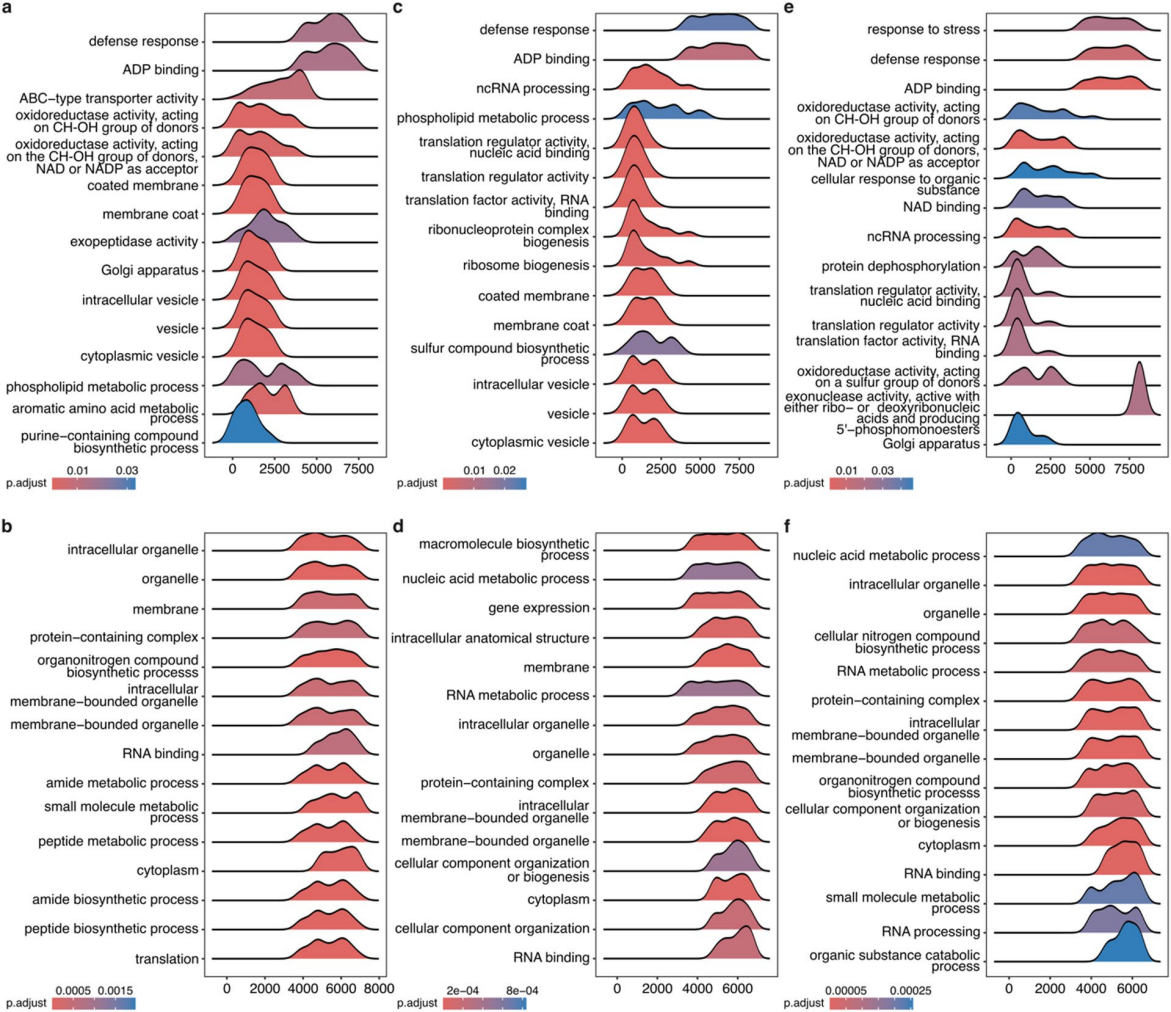
Ridge plots showing enrichment of gene ontology terms of **(a)** upregulated DEGs at 6 HAI, **(b)** downregulated DEGs at 6 HAI, **(c)** upregulated DEGs at 24 HAI, **(d)** downregulated DEGs at 24 HAI, **(e)** upregulated DEGs at 72 HAI, and **(f)** downregulated DEGs at 72 HAI. The x-axis shows the gene counts, the y-axis lists the Gene Ontology terms, the ridge indicates the density distribution of enriched genes, and the color corresponds to the adjusted p-value.

To determine the enriched gene ontology (GO) terms related to biological processes (BP), cellular components (CC), and molecular functions (MF), we performed the gene set enrichment analysis (GSEA). In comparisons of upregulated DEGs between resistant and susceptible accessions at 6 HAI (U89I6_vs_U17I6), we observed 46 total GO terms enriched, of which 23 belonged to BP, 14 to CC, and 9 to MF. At 24 HAI (U89I24_vs_U17I24), a total of 36 GO terms were found to be enriched, of which 15 were BP, 13 CC, and 8 MF. Further, at 72 HAI (U89I72_vs_U17I72), lowest number of GO terms were reported to be enriched, i.e. 17 (5 BP, 11 CC and 1 MF). In comparisons of downregulated DEGs, we observed a much larger number of GO enriched terms compared to upregulated DEGs at 6, 24, and 72 HAI in resistant vs. susceptible comparison under inoculation conditions (Table S11). The list of all the DEGs reported in all the comparison groups and their functional analysis is provided in Tables S12 and S13. The top GO terms in upregulated DEGSs reported to be related to BP were “defense response”, “aromatic amino acid metabolic process”, “phospholipid metabolic process”, “purine-containing compound biosynthetic process”; to MF were “ADP binding”, “ABC-type transporter activity”, “oxidoreductase activity, acting on CH-OH group of donors”, “oxidoreductase activity, acting on the CH-OH group of donors, NAD or NADP as acceptor”, “exopeptidase activity”; and to CC were “membrane coat”, “coated membrane”, “cytoplasmic vesicle”, “vesicle”, “intracellular vesicle”, “Golgi apparatus” at 6 HAI. Similarly at 24 HAI, enriched terms related to BP were “defense response”, “ncRNA processing”, “phospholipid metabolic process”, “ribonucleoprotein complex biogenesis”, “ribosome biogenesis”, “sulfur compound biosynthetic process”; to MF were “ADP binding”, “translation factor activity, RNA binding”, “translation regulator activity”, “translation regulator activity, nucleic acid binding”; and to CC were “membrane coat”, “coated membrane”, “cytoplasmic vesicle”, “vesicle”, “intracellular vesicle”. Further at 72 HAI, the enriched terms related to BP were “response to stress”, “defense response”, “cellular response to organic substance”, “ncRNA processing”, “protein dephosphorylation”; and to MF were “ADP binding”, “oxidoreductase activity, acting on CH-OH group of donors”, “oxidoreductase activity, acting on the CH-OH group of donors, NAD or NADP as acceptor”, “NAD binding”, “translation factor activity, RNA binding”, “translation regulator activity”, “translation regulator activity, nucleic acid binding”, “oxidoreductase activity, acting on a sulfur group of donors”, “exonuclease activity, active with either ribo- or deoxyribonucleic acids and producing 5’-phosphomonoesters”; and to CC were “Golgi apparatus”. Gene set enrichment analysis (GSEA) of upregulated DEGs revealed strong enrichment of defense-related genes across all three time points (Fig. 6). In contrast, GSEA of downregulated DEGs showed enrichment for terms associated with “organelle,” “membrane,” “RNA binding,” “cytoplasm,” “intracellular organelle,” “protein-containing complex,” “intracellular membrane-bounded organelle,” and “membrane-bounded organelle” at each of the three time points (Fig. 6, Table S13).

### Differential expression of candidate resistance genes

To identify the genes involved in *Pt* triggered disease resistance in PI 554389, we investigated the DEGs identified between treated and mock samples of the resistant accession at 6, 24, and 72 HAI. The analysis revealed two R genes, one containing LRR domain and second with kinase domain, that were significantly upregulated in response to *Pt* attack at 6 HAI. The candidate genes, viz., “AeUmb.PI554389.v2.4U.G0216100” and “AeUmb.PI554389.v2.7U.G0935200” showed log2 fold change of 5.5 and 1.2, respectively. Notably, these genes are located on the distal ends of Chr4U and 7U, at physical positions 66.5 Mb and 617.2 Mb, respectively. The strong early upregulation of these genes suggests their potential role in the early pathogen recognition and activation of immune response. These candidate R genes warrant further investigation through functional validation and could be important genes for resistance breeding to improve cultivated wheat.

## Discussion

Several diploid and polyploid wild wheat relatives (WWRs) have been utilized to enhance genetic diversity and develop disease-resilient wheat cultivars. *Aegilops umbellulata*, a diploid WWR, possesses substantial genetic potential for improving stress tolerance in wheat^16,17^. Continued advancements in genomic resources will further accelerate trait discovery and shorten the timeline for incorporating these beneficial traits into cultivated wheat varieties. Recent improvements in genome annotation tools have greatly enhanced the accuracy and precision of gene model predictions^21^. In this study, we used BRAKER3^21^,a fully automated genome annotation pipeline that integrates GeneMark-ETP, AUGUSTUS and TSEBRA, utilizing both RNAseq and IsoSeq data – to improve the annotations of the existing *Ae. umbellulata* acc. PI 554389 reference genome^17^. The revised annotation showed fewer fragmented and mono-exonic genes and higher CDS and exon completeness, consistent with well annotated plant genomes, where genuine protein coding genes tend to be multi-exonic and longer than transposable element fragments or pseudogenes^23^. Together with the 98% BUSCO completeness score, these metrics collectively support the v2 annotation as a higher confidence and a more biologically relevant gene set. Furthermore, the identification of 2310 RGAs across the genome (enriched on chromosomes 2U, 4U, and 7U) underscores the genetic potential of *Ae. umbellulata* as a valuable reservoir for disease resistance. The distribution of RGAs across chromosomes revealed that Chr4U, despite its smaller size, harbored the second highest RGA density after Chr7U. This non-uniform distribution and the clustering of RGAs towards the distal ends of chromosomes is consistent with the patterns observed in other *Triticeae* species, where resistance genes are known to accumulate in gene rich, recombination-active regions^24^. These patterns reflect the evolutionary pressure to retain or expand the immune gene diversity in specific chromosomal regions^25^. The predominance of RLK and CNL classes of RGAs and higher RGA count compared to both *Ae. tauschii* and *T. aestivum*^22^, suggests a unique and expanded set of innate immune receptors in *Ae. umbellulata*. This reinforces the status of this WWR as an important tertiary gene pool species for wheat improvement, particularly in the context of expanding resistance diversity against rapidly evolving pathogens.

To explore the functional landscape of resistance, we conducted a transcriptomics based differential gene expression analysis of two contrasting *Ae. umbellulata* accessions, following inoculation with a highly virulent *P. triticina* race (TNBJS). The phenotypic evaluation of *Ae. umbellulata* accessions PI 554389 and PI 554417 against TNBJS showed their contrasting disease response. The hypersensitive flecking response observed in PI 554389 is characteristic of gene-for-gene mediated resistance and suggests active engagement of early pathogen recognition and defense signaling^26^. The RNAseq profiling was used to examine differentially expressed genes between the contrasting accessions. PCA and clustering analyses confirmed the consistency among biological replicates and the clear separation between genotypes and treatment groups. The differential gene expression analysis across different time points reveled a dynamic transcriptional response to *Pt* infection. At 0 h, a substantial number of DEGs were detected between resistant and susceptible accessions, prior to inoculation. These differences most likely represent constitutive transcriptional variation arising from the underlying genetic divergence between the two accessions. The number of DEGs peaked at 6, 24, and 72 HAI in both the accession but with striking differences in DEG patterns between the treated and mock samples. Notably, the resistant accession exhibited more DEGs at 6 HAI, suggesting a rapid and robust activation of defense pathways, while the susceptible accession showed more transcriptional changes at the later stages of infection, potentially reflecting a delayed or ineffective response. These findings are consistent with previous reports where early and strong gene activation correlated with effective resistance^27–29^. To validate the RNAseq results, we performed qRT-PCR on a subset of eight randomly picked genes across four key treatment comparison groups. The qRT-PCR data showed strong concordance with the RNAseq log_2_ fold changes, which confirms the reliability of the expression profiles. This cross validation reinforces the robustness of the transcriptomic analysis and supports the biological conclusions drawn from the DEG datasets.

A functional enrichment analysis of the DEGs revealed that the upregulated genes in the resistant accession were predominantly associated with defense related GO terms. These terms reflect known immune processes including pathogen recognition, signal transduction, and the activation of vesicle-mediated secretion of defense molecules. The enrichment of translation-related and ribosome biogenesis terms at 24 HAI suggests a coordinated effort to sustain protein production during the defense response. In contrast, downregulated DEGs were enriched for cellular structural and metabolic terms, indicating potential reallocation of cellular resources toward defense mechanisms. These findings imply a complex and multi-layered defense response activated in response to *Pt* infection.

Importantly, we report the identification of two candidate R genes, “AeUmb.PI554389.v2.4U.G0216100” and “AeUmb.PI554389.v2.7U.G0935200”, that were significantly upregulated at 6 HAI in the resistant accession, PI 554389. Their early and strong induction (log_2_FC of 5.5 and 1.2, respectively) and distal chromosomal localization make them strong candidates for the functional R gene validation. Both candidate genes identified in this study encode canonical resistance-related domains, LRR and kinase, indicating their potential roles in distinct layers of immune signaling. The LRR domain is typically associated with effector recognition, functioning either within intracellular NLR receptors or as part of extracellular receptors that confer recognition specificity. In contrast, the kinase-containing gene may participate in early phosphorylation cascades that amplify immune signaling downstream of recognition, consistent with the established functions of receptor-like kinases (RLKs) in pattern-triggered immunity. The strong early induction of both genes at 6 HAI suggests their involvement in the initial pathogen perception and signaling events that lead to hypersensitive response and restriction of *P. triticina* infection. From an applied standpoint, these candidate R genes represent promising targets for wheat improvement efforts aimed at enhancing durable disease resistance. After functional validation through approaches suchas virus-induced gene silencing (VIGS), transient expression assays, or transgenic complementation, these genes could serve as prime candidates for introgression into hexaploid or synthetic wheat lines to enhance disease resistance^30^. Their early activation of these genes makes them strong candidates for exploring broad-spectrum and durable resistance mechanisms, with potentially applicationsin breeding programs aimed at mitigating the impact of leaf rust epidemics.

In conclusion, the improved genome annotation of *Ae. umbellulata* provides a more accuarate and comprehensive set of gene models, strengthening the foundation for future functional and comparative genomic studies in wild wheat relatives. Our transcriptomic analyses revelaed dynamic and genotype-specific responses to *P. triticina* infection, offering valuable insights into molecular mechanisms underlying leaf rust resistance. Importantly, we identified two early induced candidate resistance genes containing kinase and LRR domains, which represent promising targets for functional validation, marker development, and introgression into cultivated wheat to enhance durable resistance.

## Materials and methods

### Plant material and disease phenotyping

*Aegilops umbellulata* accessions showing highly resistant (PI 554389) and susceptible (PI 554417) responses to several leaf rust races^17^ were used in the present study. These two accessions were originally collected from Turkey and the seeds were obtained from the USDA-ARS Germplasm Resources Information Network (GRIN). Seeds from each accession were sown in the AES growth chamber under biosafety level 2 (BSL 2) at the North Dakota State University, Fargo, North Dakota, US. The growth chamber was adjusted to 16 h (hrs) of light with day and night temperatures of 22 °C and 18 °C, respectively. Seedlings at two leaf stage were evenly inoculated with the most virulent race of *Puccinia triticina* (i.e., TNBJS) in the USA, by suspending fresh urediniospores at a concentration of 1 × 10^6^ spores mL^− 1^ in the mineral oil (viz., SOLTROL-170). An air pump with regulated pressure was used for the inoculation. Additionally, the uninoculated control group of each accession was evenly sprayed with SOLTROL-170 only and were considered as mock treatments. After volatilization of mineral oil, plants were kept in dark humid chambers for 16–18 h adjusted with 20 °C overnight and then moved back to the greenhouse. The infection-type (IT) assessment for leaf rust was conducted using 0–4 scale, where ‘0’ = no visible uredinia, ‘;’ = hypersensitive flecks, ‘1’ = small uredinia with necrosis, ‘2’ = small to medium-sized uredinia with green islands and surrounded by necrosis or chlorosis, ‘3’ = medium-sized uredinia with or without chlorosis, ‘4’ = large uredinia without chlorosis. The variations in prominent disease IT represented using “+” or “-” signs^31^.

### Sample collection, RNA extraction and sequencing

Leaf samples were collected from both inoculated and uninoculated (mock) plants at four time points (viz., 0, 6, 24, and 72 HAI). The 14 samples were planted in three replications with a total of 42 samples for the experiment. Upon sample collection, the leaf tissues were immediately frozen in the liquid nitrogen and were stored in the freezer (-80 °C) until RNA extraction. Leaf samples were ground to a fine powder using liquid nitrogen in a pestle and mortar and the total RNA was extracted using the Qiagen RNeasy Plant Mini Kit (Catalog # 74904). For DNase treatment, TURBO DNA-free kit was used following the manufacturer’s instructions, to remove any genomic DNA contamination. The total RNA was subjected to quality and quantity assessment using Nanodrop spectrophotometer and Bioanalyze 2100 (Agilent). For library preparation, a total of 1 µg RNA was used. The library preparation and paired-end RNA sequencing was outsourced using from Novogene (www.novogene.com).

### Quality check of transcriptomics data

To ensure the strandedness of the generated data, we used infer_experiment.py (v5.0.2) script from RSeQC package (https://rseqc.sourceforge.net/#usage-information)^32^, mapped a subset of reads on the reference genome of *Ae. umbellulata* accession PI 554389 using HISAT2 (v2.2.0)^33^, and ran the infer_experiment.py script to determine the standedness. Our RNAseq data was found to be unstranded. Further, for quality assessment, adapter trimming, and low quality read filtering, we used fastp (v0.23.4)^34,35^.

### Re-annotation and resistance gene analogs (RGAs) prediction

We used BRAKER3^21^ pipeline to improve the accuracy of gene models of reference genome of *Ae. umbellulata* accession PI 554389. The short reads generated in this study and long reads from our previous PacBio Iso-seq^17^ were used to re-annotate the gene models. Further, the gene models were named by following a common gene naming convention, i.e., species name, accession (cultivar) name, annotation version, chromosome number, and gene number. For alternative transcripts, a numeric digit has been added for each transcript after the gene name. An example of a gene name is AeUmb.PI554389.v2.1U.G0517000 and of corresponding transcript is AeUmb.PI554389.v2.1U.G0517000.1. Further, the gene models were classified into high-confidence (HC) and low-confidence (LC) categories based on sequence similarity (BLAST) to the UniProt TrEMBL dataset for Magnoliopsida and the Triticeae repetitive element database (TREP). HC gene models were defined as those with significant homology to Magnoliopsida TrEMBL entries (E-value < 1e − 10), exhibiting > 66% amino acid identity and a subject/query length difference < 25%. Gene models that did not meet these thresholds or lacked external support were designated as LC. Additionally, any gene model with a significant hit (E-value < 1e − 10) to the TREP database was classified as LC to account for potential transposable element–related sequences.For BUSCO assessment of these gene models, we used POALES dataset^36^. We used the docker image of RGAugury^37^ (https://bitbucket.org/yaanlpc/rgaugury/src/master/) with the default parameters and databases such as pfam and interproscan, to predict RGAs in the reannotated genome.

### RNAseq mapping and quantification

We used STAR (v2.7.6a) RNA-seq aligner^38^ for alignment of cleaned RNA-seq reads to the *Ae. umbellulata* accession PI 554389 reference genome^17^. First, we used “--runMode genomeGenerate” to create genome index for the reference genome. Then, we mapped all the samples separately to the reference genome using STAR. Further, gene level read counts were quantified from mapped data using featureCounts (v2.0.6)^39^. StringTie (v2.2.1) was used for gene quantification^40^.

### Differential gene expression analysis

After read quantification, DESeq2 (v1.44.0) package^41^ was used for the differential expression (DE) analysis in R (v4.4.0). The output from featureCounts in R along with the meta data for all the samples was used for this analysis. The genes with low read counts (less than 10 reads across all the samples) were filtered out. The inbuilt read count normalization capability of DESeq2 was used to identify the differentially expressed genes (DEGs) by fitting the negative binomial model. For our study, we used multiple factors, such as genotypes (Resistant vs. Susceptible), treatment (inoculated vs. mock), and time (0, 6, 24, and 72 HAI) for hypothesis testing as given in Table S8. To control the false discovery rate (FDR), Benjamini and Hochberg (BH) method was used. We considered genes with adjusted p-values less than 0.05 and log_2_FC greater than 1 as DEGs. For any data wrangling and plotting, we used R packages, dplyr (v1.1.4), tidyverse (v2.0.0) and ggplot2 (v3.5.1). Further, we used pheatmap (v1.0.12), VennDiagram (v1.7.3), UpSetR (v1.4.0), EnhancedVolcano (v1.22.0) to draw heatmaps, venn diagrams, upset plots and volcano plots, respectively. For principal component analysis, variance stabilizing transformation in DESeq2 was used.

### Gene set enrichment analysis

For functional annotations, Interproscan (v5.63-95.0) was used with “all appl” to retrieve the gene ontology (GO) terms^42^. To build an organism database (OrgDb) of GO terms, we used AnnotationForge (v1.46.0), GO.db (v3.19.1), AnnotationDbi (v1.66.0). This “org.Aumbellulata.eg.db” database has been deposited to Figshare (DOI: 10.6084/m9.figshare.30513785). For further GSEA, clusterProfiler (v4.12.2) was used. We preformed (GSEA) on DE genes (separately on up-regulated and down-regulated genes), using “gseGO” from clusterProfiler with these parameters: “OrgDb = org.Aumbellulata.eg.db, keyType = “GID”, ont = “ALL”, minGSSize = 10, maxGSSize = 500, pAdjustMethod = “BH”, pvalueCutoff = 0.05, verbose = TRUE, nPermSimple = 10000”. For ploting, we used ggplot2 (v3.5.1) package from R.

### Quantitative real time reverse transcription-PCR (qRT-PCR) analysis

We used ~ 1 µg RNA to synthesize first-strand cDNA using SuperScript™ III First-Strand Synthesis System (Catalog #18080051). For qRT-PCR validation, eight DEGs were selected. The primers were designed using PrimerQuest tool (https://www.idtdna.com/pages/tools/primerquest) using “2 primers; for use with intercalating dyes” option and were synthesized from Integrated DNA Technologies, Inc. (Table S10). The total reaction volume of 6 µl included 3 µl SYBR Green Supermix, 2 µl of diluted cDNA (1:10), and 1 µl Forward and Reverse primers (833nM). We used SsoAdvanced Universal SYBR Green Supermix (Catalog #1725271) on a BioRad CFX384 Real-Time System using manufacturer’s instructions. For internal controls, we used GAPDH, a housekeeping gene. The ∆∆ct method was used for expression data analysis^43^.

## Supplementary Information

Below is the link to the electronic supplementary material.

Supplementary Material 1

Supplementary Material 2

Supplementary Material 3

Supplementary Material 4

## Data Availability

The raw Illumina data produced in this study is deposited in NCBI SRA database with BioProject accession number PRJNA1316474 and can be accessed at https://www.ncbi.nlm.nih.gov/sra/PRJNA1316474.
