# Supplementary material for: Improved reannotations of Aegilops umbellulata (PI 554389) genome and transcriptomics data provide candidates for leaf rust resistance for wheat improvement
Source: Sci Rep. 2025 Dec 29;15:44930. doi: 10.1038/s41598-025-29241-6 (PMC12749518; doi:10.1038/s41598-025-29241-6)
Supplement: Supplementary file 4 — Supplementary Material 4 [file 41598_2025_29241_MOESM4_ESM.pdf]

Supplementary Figures

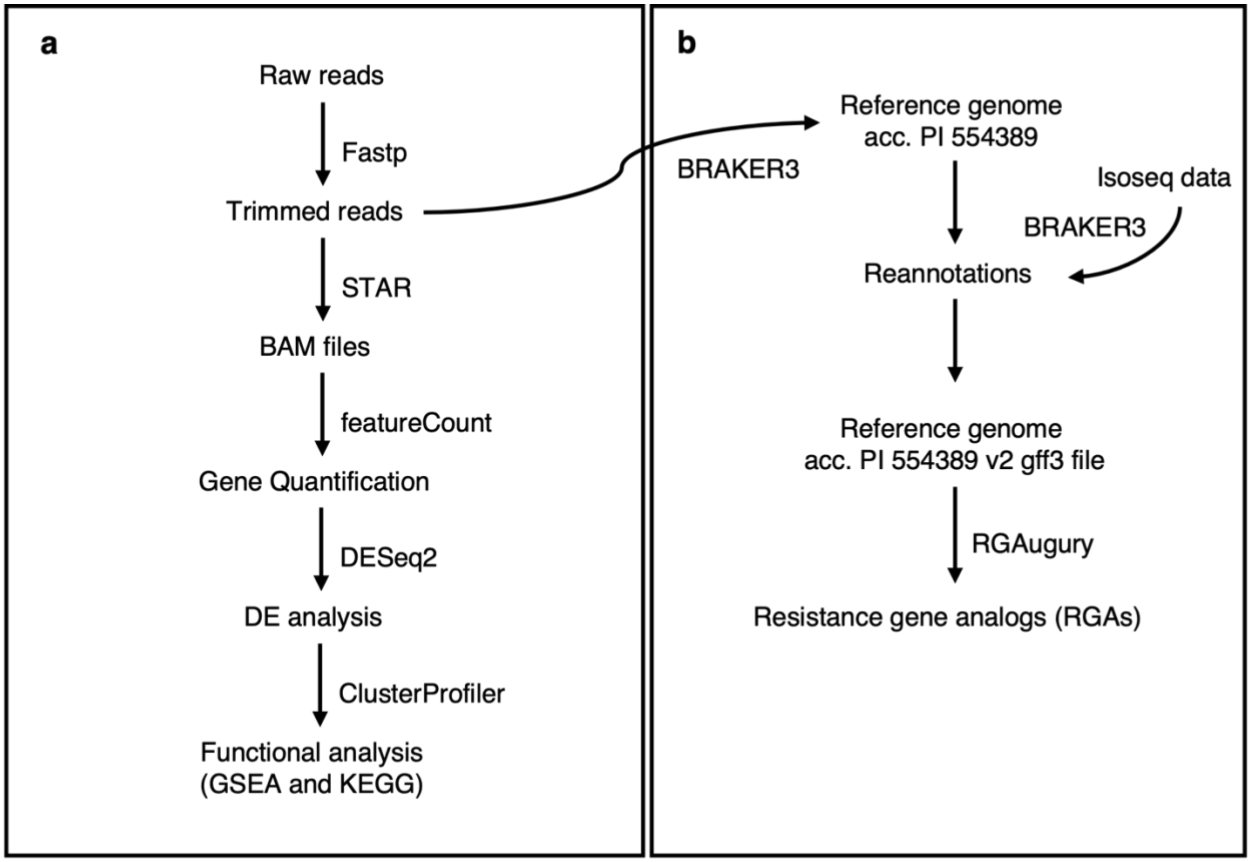

Figure S1: Data analysis workflow used for the RNAseq analysis **(a)**, and reannotations and resistance gene analogs (RGAs) identification **(b)**.

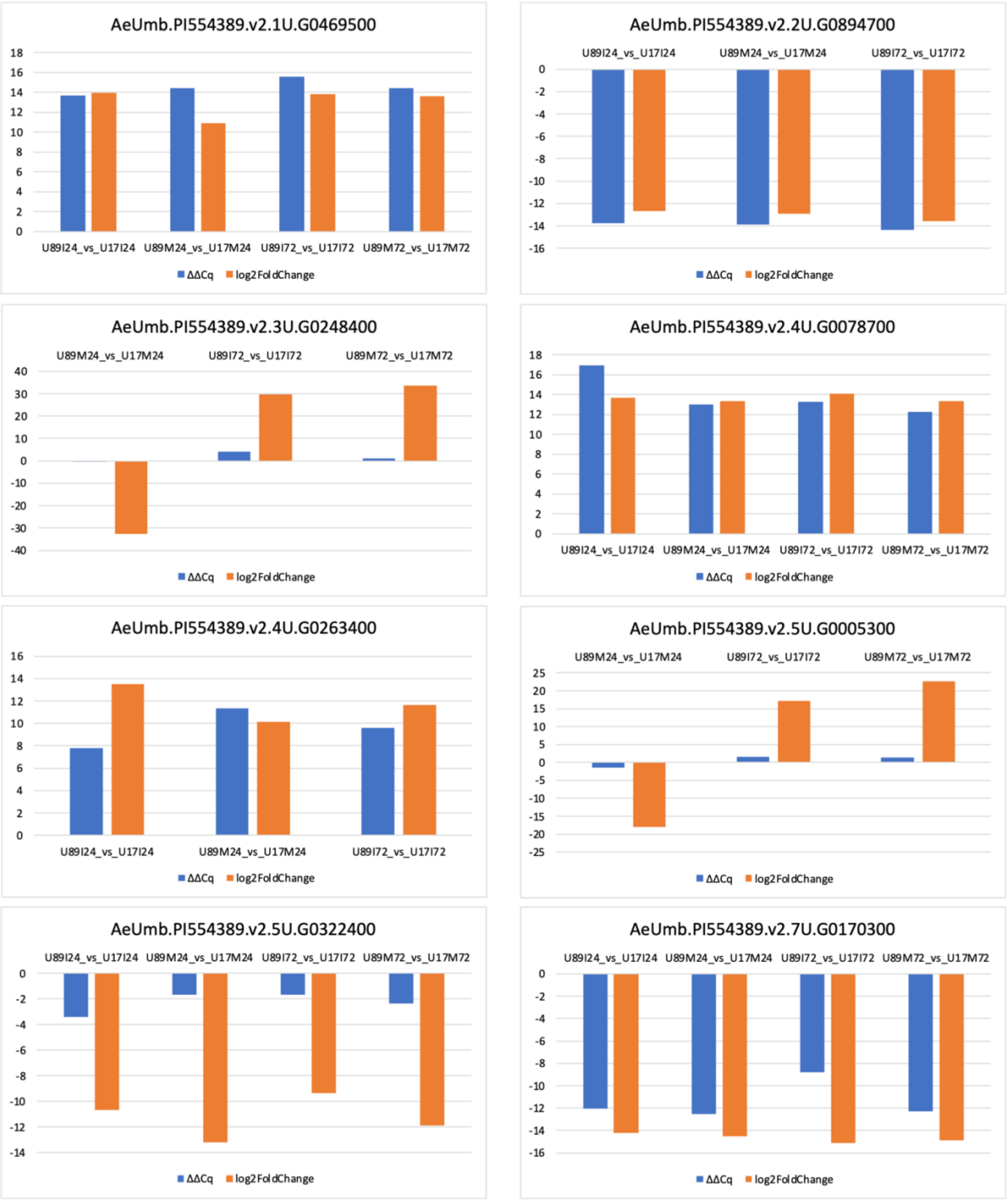

Figure S2: Bar plot showing the comparison of gene expression levels determined by qRT-PCR and RNA-seq for selected genes across four treatment conditions: U89I24_vs_U17I24, U89M24_vs_U17M24, U89I72_vs_U17I72, and U89M72_vs_U17M72.
